# Shampoo-Clay Heals Diaper Rash Faster Than Calendula Officinalis

**Published:** 2014-06-15

**Authors:** Mohsen Adib-Hajbaghery, Mansoreh Mahmoudi, Mahdi Mashaiekhi

**Affiliations:** 1 Trauma Nursing Research Center, Kashan University of Medical Sciences, Kashan, IR Iran; 2 Department of Medical-Surgical Nursing, Faculty of Nursing and Midwifery, Arak University of Medical Sciences, Arak, IR. Iran; 3 Department of Pediatrics, Emam Khomeini Hospital, Khomein, Markazi Province, IR Iran

**Keywords:** Diaper Rash, Calendula, Infant, Wound Healing

## Abstract

**Background:**

Diaper rash is one of the most common skin disorders of infancy and childhood. Some studies have shown that Shampoo-clay was effective to treat chronic dermatitis. Then, it is supposed that it may be effective in diaper rash; however, no published studies were found in this regard.

**Objectives:**

This study aimed to compare the effects of Shampoo-clay (S.C) and Calendula officinalis (C.O) to improve infantile diaper rash.

**Patients and Methods:**

A randomized, double blind, parallel controlled, non-inferiority trial was conducted on 60 outpatient infants referred to health care centers or pediatric clinics in Khomein city and diagnosed with diaper rash. Patients were randomly assigned into two treatment groups including S.C group (n = 30) and C.O group (n = 30) by using one to one allocation ratio. The rate of complete recovery in three days was the primary outcome. Data was collected using a checklist and analyzed using t-test, Chi-square and Fisher’s exact tests and risk ratio.

**Results:**

Totally, 93.3% of lesions in the S.C group healed in the first 6 hours, while this rate was 40% in C.O group (P < 0.001). The healing ratio for improvement in the first 6 hours was 7 times more in the S.C group. In addition, 90% of infants in the SC group and 36.7% in the C.O group were improved completely in the first 3 days (P < 0.001).

**Conclusions:**

S.C was effective to heal diaper rash, and also had faster effects compared to C.O.

## 1. Background

Diaper rash is one of the most common skin disorders of infancy and childhood (1). It is characterized by an acute inflammatory reaction of the skin around diaper (2). This disorder might be due to frequent and prolonged contact of the skin with urine, stool and moisture, and exacerbated by candidiasis infection and abrasion (2, 3). Erythema, flaking, papules and lesions in buttocks, thighs, scrotum and mons pubis are among the frequent signs and symptoms of diaper rash (4). This disorder commonly occurs in the age of 9 to 12 months old (5), and its prevalence has been reported to be around 7 to 35 and even 50 percent (6). The prevalence of diaper rash among infants is reported to be 75% in the United States (US), 87% in Japan, 15% in Italy (7), and 34.9% in Iran (8). The onset of this disorder commonly occurs within the first third to twelfth weeks of life, but its peak is in the first seventh to twelfth weeks (9).

Routine treatment options for diaper rash include changing the diaper and washing the genital area frequently, applying Vaseline or zinc oxide, and corticosteroids (10, 11). Calendula officinalis (C.O) may also be effective in non-complicated cases (12). C.O has antiseptic and anti-inflammatory properties (13). Shampoo-clay (SC) (also called Bentonite) is basically a kind of mineral in the form of aluminum phyllosilicates and its powder is so fine, odorless, white to grey, or yellow or pink. This powder can absorb high amounts of water and is used as protector and water absorbent (14). Studies have shown that SC was effective in the treatment of chronic dermatitis of the hands (15). Then, it is supposed that it may be effective in the treatment of other skin disorders and diaper rash. 

## 2. Objectives

This study was aimed to compare the effects of SC and C.O on healing of infantile diaper rash.

## 3. Patients and Methods

The present study was a randomized, double blind, parallel, controlled, non-inferiority trial conducted during February and March 2013 on 60 outpatient infants referred to health care centers or pediatric clinics in Khomein city. All patients were visited by a general practitioner or a pediatrician and diagnosed with diaper rash. Infants with age range of 1 to 24 months, having a medical diagnosis of mild (redness, abrasion, skin atrophy) to medium (redness with papules, abrasion, skin atrophy) diaper rash, not having infantile eczema, diarrhea, and urinary tract infection, not having fungus dermatitis and not using corticosteroids for present lesions were enrolled in the study. However, using corticosteroids, not following the program, exacerbation of lesions and developing diarrhea during the study were considered as exclusion criteria.

Sample size was calculated based on data obtained from a pilot study designed similar to the main study and seven infants were assigned to each group. Then, five infants in C.O group (0.71%) and seven infants in SC group (100%) completely improved in three days. Then, 28 samples were estimated for each group based on the following formula (α= 0.05, β = 0.80, p1 = 1.0, p2 = 0.71). For more certainty and through one to one allocation ratio, 30 samples were randomly selected and assigned into each treatment including SC (n = 30) and C.O (n = 30). The primary outcome was the rate of complete recovery in the first three days. To begin the study, the researcher talked to the parents, explained the study process, took their agreement (by taking the written informed consent), and then took an initial skin test on the infant’s arm to ensure the absence of allergic reaction. If any allergic reactions were not observed 20 minutes after applying a fingertip of SC and C.O on the infant’s arm, infants were allowed to enter the study. Then, the samples were assigned randomly in SC group or in the C.O group (if redness or any other allergic reactions were not observed).

To produce SC 50%, the mineral was squashed and powdered, sterilized on oven, mixed with water (50 grams SC was mixed with 50 milliliters of distilled water) and poured in sterile cans and prepared in the form of SC 50% cream in 30 grams cans by a pharmacist. Also, 30 grams of C.O 1.5% cream (produced by Dineh Company) was poured in the similar 30 grams sterile cans. C.O and SC were prepared in cans with similar shape and weight and then all cans were coded by the pharmacist.

A checklist was prepared through literature review and then its content validity was assessed by 10 faculty members of Kashan and Arak Universities of Medical Sciences. The reliability of the checklist was assessed through inter-observers reliability. Therefore, the second researcher and a trained co-researcher used the checklist for ten patients and a rate of agreement of 0.93 was calculated. The checklist included questions about the infants’ age, gender and weight, and the mother's education level, age and job. Also there were questions on the infants’ type of feeding, history of diaper rash, the severity of the disorder, drugs used in previous episodes of the disease and the frequency of changing diaper in a day. There was also a table for recording the onset of healing in the first six hours (yes, no), and time of complete healing (1, 2, 3, more days after starting the treatment).

The second researcher completed checklists for each infant and then the physician (un-aware of the cans cods) visited infants and prescribed one of the two creams randomly. A can contained one of the drugs was given to the mother of each infant and they were told that additional drugs would be prescribed in the next visit if necessary. Mothers were un-aware of the type of drug. The second researcher educated all mothers to apply the cream on the affected area four times a day. All mothers were taught:

1. To wash the affected area with lukewarm water and dry it with a clean cotton towel.
2. To spread the prescribed cream on the affected area as it covers one centimeter over the lesions borders, then diaper the baby, and repeat this every 4-6 hours or more if needed.

All mothers were advised not to apply any other materials or medications on the affected areas.

The second researcher followed the process of administrating the cream, following the treatment program and the effect of treatment three times a day by phone. Mothers monitored the onset of recovery and reported the researcher when the researcher called them. In addition, every other day (up to 3 times), the infants were visited and assessed both by the researcher and physician and additional cream was given to the mothers if needed. The effect of treatment (healed or not) was documented based on the physician's decision.

### 3.1. Ethical Considerations

The Research Council and the Human Research Ethics Committee of Kashan University of Medical Sciences approved the study protocol. In addition, parents of all babies were informed about the design of study and assured about the data confidentiality, safeness of the study and their right not to participate. They also signed a written informed consent. The parents were also assured that their baby would be under close and frequent observation of the research team for any probable side effect or delay in recovery. We also observed all ethical issues in accordance with the latest version of the Declaration of Helsinki.

### 3.2. Data Analysis

The data was analyzed by using SPSS software, version 16.0. Independent sample T-test was used to compare quantitative variables (i.e. age, weight), and Chi-Square, Risk ratio, and Fisher’s exact test were used to compare qualitative variables between the two groups such as healing.

## 4. Results

**Figure 1.**
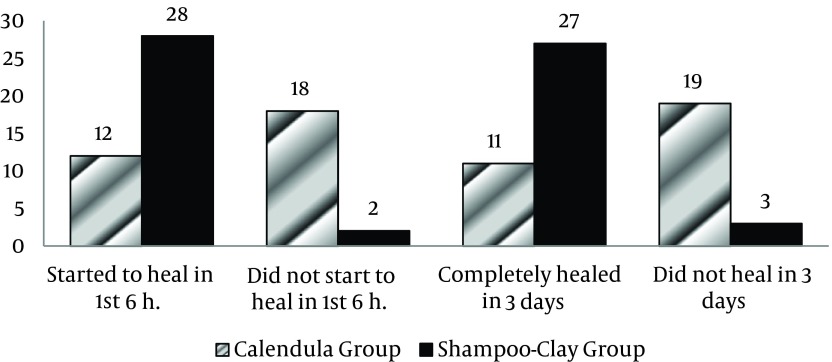
Healing Rates in the Two Groups

Demographic characteristics of the study groups are presented in Table 1 and no significant differences were observed between the two groups in term of these variables (Table 1). Also, no significant differences were observed between the two groups in terms of nutrition type (P=0.78), previous history of diaper rash (P=0.30). Moreover, the two groups were not significantly different in terms of mothers characteristics such as age (P = 0.52), job (P = 0.38) and education level (P = 0.84). Overall, 28 infants (93.3%) in SC group and 24 infants (40%) in C.O group started to heal in the first 6 hours after the treatment. (P < 0.001) (Figure 1). Moreover, the risk ratio for the healing in the first 6 hours was 7 times more in the SC group than C.O group (CI = 1.85, 26.47). Furthermore, 27 cases (90%) in the SC group and 11 cases (36.7%) in the C.O group were completely healed in the first 3 days (P < 0.001) (Figure 1). Besides, the risk ratio for the complete healing in the first three days was 5.21 times more in the SC group than the C.O group (CI = 1.78, 15.2).

**Table 1.** Personal Characteristics of the Infants and Their Mothers ^a^

	Shampoo-clay	Calendula officinalis	Test Results		
			T-Test	P Value	X^2^
**Age, m**	6.88 ± 5.62	5.68 ± 4.63	-0.88	0.38	
**Weight, kg**	6.94 ± 2.59	6.60 ± 2.66	-0.49	0.93	
**Gender**			-	0.78	0.07
Female	21 (70)	20 (66.7)			
Male	9 (30)	10 (33.3)			
**Severity of Diaper Rash**				0.11	2.44
Mild	16 (53.3)	10 (34.5)			
Moderate	14 (46.7)	19 (65.5)			
**The frequency of changing diaper a day**	5.66 ± 3.03	5.35 ± 1.87	-0.65	0.15	-

^a^ Data are presented in No. (%) or Mean ± SD.

## 5. Discussion

This study aimed to compare the effects of SC and C.O to improve infantile diaper rash. Findings showed that the onset of healing in the first 6 hours was seven times more in the SC group than the C.O group. Moreover, complete healing in the first 3 days was more than five times in the SC group. Emami-Razavi et al. in a study on rats, reported that SC was effective in healing of skin wounds (16). Moreover, Fowler assessed the effect of a moisturizer cream containing SC on improvement of chronic dermatitis of the hands on human subjects and reported that it could accelerated the improvement of chronic dermatitis in an 8 week period (15).

No studies were found regarding the effectiveness of SC to improve diaper rash. However, some studies assessed the effects of herbal or complementary products such as honey, olive oil and beeswax on this disorder and reported positive effects (17, 18). One study also compared the effects of C.O and Betamethasone to prevent acute radiation dermatitis and reported no significant difference between the two medications (19). Also, Panahi et al. compared the effects of *Aloe vera* and C.O on children younger than three years old with diaper rash and reported that C.O was more effective than *Aloe vera* (12). However, the present study showed that SC was not only more effective than C.O to improve diaper rash, but also accelerated the healing.

In conclusion, the present study showed that SC was effective in healing diaper rash. It also had faster effects compared to C.O. These advantages might be related to anti-inflammatory, antibacterial, water absorbing and skin protecting properties of this traditional product. Moreover, no side effects were observed in the two groups. However, the study sample in the present study was small, and it is recommended to conduct similar studies with larger sample size. Besides, the present study was conducted on infants with mild to moderate diaper rash, so it is recommended to conduct multicenter studies on severe cases. 
